# The Use of Tag Questions in Person-centered Communication

**DOI:** 10.1093/geroni/igaa057.3313

**Published:** 2020-12-16

**Authors:** Shalane Basque, Marie Savundranayagam, Maren Kimura, Kristine Williams

**Affiliations:** 1 Western University, London, Ontario, Canada; 2 University of Kansas, Lawrence, Kansas, United States

## Abstract

Tag questions are imperative, declarative, exclamative or interrogative statements that have been modified to include a question (e.g.., It is hot out, isn’t it?). Tag questions have been characterized as elderspeak because it suggests an expected response from the person with dementia, thus limiting his/her ability to make a decision independently. However, tag questions serve multiple functions in conversation. There is limited research on the multidimensional nature of tag questions in conversations between formal caregivers and their clients with dementia. Accordingly, the purpose of this study was to investigate the functions of tag questions used by formal caregivers in utterances coded as person-centered. Conversations (N= 87) between formal caregivers and a simulated person with dementia were video-recorded during a 5-minute care interaction involving morning care. Caregivers’ utterances were coded for the use of the following types of person-centered communication: recognition, negotiation, facilitation, and validation. During secondary data analysis, the person-centered utterances were analyzed for the use and function of tag questions. Conversational analyses revealed two broad functions of tag questions: gather information and facilitate a desired action. Tag questions used to gather information included the following specific functions: acknowledge response, establish common ground, state fact or opinion, initiate topic, conversational joking, state what is being done and questions. Tag questions that facilitated a desired reaction included the following specific functions: offers, advice and suggestions and requests and commands. Findings from the current study reveal that tag questions are not exclusively elderspeak and can be used to illicit conversation.

